# Building the Evidence Base for the Prevention of Raw Milk-Acquired Brucellosis: A Systematic Review

**DOI:** 10.3389/fpubh.2020.00076

**Published:** 2020-03-13

**Authors:** Shakirat A. Adetunji, Gilbert Ramirez, Allison R. Ficht, Ligia Perez, Margaret J. Foster, Angela M. Arenas-Gamboa

**Affiliations:** ^1^Department of Veterinary Pathobiology, Texas A&M University, College Station, TX, United States; ^2^School of Public Health, Texas A&M University, College Station, TX, United States; ^3^College of Medicine, Texas A&M University Health Science Center, Bryan, TX, United States; ^4^Department of Student Life Studies, Texas A&M University, College Station, TX, United States; ^5^Medical Sciences Library, Texas A&M University, College Station, TX, United States

**Keywords:** brucellosis, evidence, gaps, raw milk, systematic review

## Abstract

**Background:** The scientific evidence of the health risks associated with the consumption of raw milk has been known for a long time. However, less clear is the impact of acquiring infectious diseases from raw milk consumption in the United States (US) due to incomplete reporting of cases and the complex factors associated with the sale and consumption of raw milk. Investigations of this current study focused on human brucellosis, one of the infectious diseases commonly acquired through the consumption of raw milk and milk products, and which continues to be a public health threat worldwide.

**Methodology:** A qualitative systematic review of the sources of opinions that contribute to the increased trend of raw milk sales and consumption in the US was conducted.

**Results:** Interestingly, opinions about the sale of raw milk and/or the benefits arising from its consumption varied by US region, with the proportion of messages supporting raw milk consumption being highest in the Northeast compared to other US regions. Several evidence gaps and factors that possibly contribute to the increased prevalence of raw milk-acquired brucellosis were identified including inadequate monitoring of the raw milk sales process and lack of approved diagnostic methods for validating the safety of raw milk for human consumption.

**Conclusions:** The unavailability of data specifying brucellosis cases acquired from raw milk consumption have precluded the direct association between raw milk and increased brucellosis prevalence in the United States. Nevertheless, the evidence gaps identified in this study demonstrate the need for intensified surveillance of raw-milk acquired infectious diseases including human brucellosis; establishment of safety and quality control measures for the process of selling raw milk; and design of an effective strategy for the prevention of raw milk-acquired infectious diseases including brucellosis. Overall, for the first time, this study has not only shown the gaps in evidence that require future investigations, but also, variations in the perception of raw milk consumption that may impact disease acquisition in different US regions.

## Introduction

Brucellosis, one of the world's most common bacterial zoonosis, is an ancient disease that dates as far back as the 1800s (1–3). The discovery of the disease was by David Bruce, a military physician stationed in the island of Malta in the 1880s. During this period, Bruce noted the increased manifestation of a disease characterized by undulant fever and joint pains that debilitated many British soldiers. Autopsy of the deceased soldiers led to the recovery of the causative organism from the spleens, livers, and kidneys. To confirm that the recovered organism was the cause of the disease, Sir Bruce reproduced the infection in monkeys using bacterial cultures from the spleen of infected soldiers. A common practice at that time in Malta was the consumption of fresh raw goat milk (4). Raw goat milk was later confirmed to be the source of the bacteria, and the first consideration of brucellosis as a zoonosis arose from the isolation of its causative agent, *Brucella melitensis*, from goat milk (2, 3, 5). Subsequently, the prohibition of goat milk and cheese in military establishments led to a significant reduction of the disease incidence among the soldiers of Malta (2).

In the United States, brucellosis was reported as early as the 1900s where 29 cases of brucellosis (*B. melitensis*) were reported in Houston among Mexican immigrants that had consumed goat cheese before the onset of their symptoms. Additionally, between 1965 and 1978 in the US, over 3,000 cases of brucellosis were reported, and 4% of these cases were attributed to raw dairy products from Mexico, predominantly from the consumption of fresh cheese from unpasteurized goat milk (2, 6).

Although, brucellosis incidence has been attributed to varying factors, the consumption of unpasteurized dairy products accounts for a large number of cases, particularly in endemic countries such as Asia, Middle East, Africa, Central and South America (7–9). Further, in non-endemic countries, brucellosis has also been reported to occur after travel to, and consumption of raw dairy products in endemic countries (10).

In developed countries, the emerging interests in natural foods and products have led to the increased preference for raw milk consumption due to its acclaimed health benefits that are believed to be destroyed upon pasteurization (11, 12). Pasteurization, a process which dates back to the 1800s, involves the heating of raw milk to a defined temperature for a specific period of time to inactivate live, disease-causing organisms such as *Brucella, Salmonella, Listeria, Campylobacter, E. coli*, amongst others that pose significant health risk to consumers (13, 14). The process has been invaluable in the improvement of the safety of milk and other food products for human consumption. Another added advantage of pasteurization is that it destroys organisms that cause food spoilage, thereby increasing shelf-life and enhancing food security in low to middle income countries (11, 14–16). The presence of harmful pathogens in milk or dairy products can occur from either a direct passage from the animal, contamination of the expressed milk by animal excreta, or unsanitary handling of the milking process (13). For many years, several outbreaks of diseases resulting from the consumption of raw dairy products have been reported to the Centers for Disease Control and Prevention (CDC) (6, 15, 17–19). In recent times, at least three cases of human brucellosis have been confirmed by the CDC resulting from an exposure to the live-attenuated vaccine strain *Brucella abortus* RB51 following the consumption of raw milk (18, 19). In the most recent outbreak, it is believed that hundreds of persons in approximately 19 states may have been exposed in connection to the consumption of raw milk from a farm in Pennsylvania (19).

Interestingly, despite the significant public health risk that raw milk presents to consumers, the sale of raw milk for human consumption is not prohibited in all states in the US (11, 14, 20). Currently, 13 states allow raw milk to be sold in retail stores, 17 states allow raw milk to be sold only on farms where the milk is produced, 8 states allow raw milk to be obtained via the cow-share program (which involves the leasing of cows to obtain a percentage of a cow's milk production), while 21 states prohibit the sale of raw milk for human consumption (6). Interestingly, outbreaks of raw milk-related diseases including brucellosis have been reported mostly in states that legalize the sale of raw milk (11, 17, 19, 21, 22).

Previous studies have highlighted the varying motivations that drive raw milk consumers including consuming food items in their pure natural forms, better tastes and flavors, the belief that pasteurization destroys the natural components of milk, support of local farmers, and lack of trust of the state government as regards regulation of safe foods for consumption (16, 21, 23).

To date, scientific validation of the health benefits of consuming raw milk is very limited, and it has been extensively demonstrated that the health risks associated with the consumption of raw milk significantly outweigh the unfounded proclaimed health benefits (6, 21, 24, 25). Additionally, information exchanged via social media and networks have been shown to influence the attitudes and decisions of consumers (16, 21, 23, 26). Currently, there is a gap in knowledge about the variables by which consumers evaluate the information exchanged in their food safety and preference conversations, or how consumers perceive the varying recommendations regarding raw milk.

Despite the significant health risks posed by the consumption of unpasteurized milk and dairy products, there is still an increased trend in the purchase and consumption of raw milk (21), which may consequently lead to an increased prevalence of raw-milk acquired brucellosis as well as other diseases in the US. In order to design a more effective approach to educate consumers on the public health risks associated with this practice, the significance of the sources of information related to the purchase and consumption of raw milk and milk products must be critically evaluated to enhance or come up with an effective strategy in the control and prevention of raw milk-acquired brucellosis. Therefore, the objectives of this report are to identify the evidence gaps for future investigations that will facilitate informed policy decision about the sale and consumption of raw milk and milk products in the US, and to systematically review the sources of information that contribute to the increased trend of raw milk sale and consumption in the US, and associate the findings with the rising prevalence of raw milk-acquired brucellosis cases in the country. Results from this current study will facilitate efforts that are necessary to enhance research into the development of innovative approaches to disseminate information about the dangers of raw milk consumption; intensify the surveillance of human brucellosis as a differential diagnosis to enable physicians to better control the disease; establish a quality control of the sales process; and highlight the significance of collecting and analyzing data about nation-wide raw milk sales, which will help to frame food safety policies for the benefit of the human population.

## Materials and Methods

### Eligibility Criteria

To systematically review public opinions about the consumption of raw dairy products in the US, potential sources of public opinion including newspapers, magazines, and newsletters were searched using the EBSCO information services. The search was restricted to the US and a span years (2012 to 2017). Information sources expressing an opinion that was neutral, supportive or against the consumption of raw milk were included in the study. Peer-reviewed scientific publications, reports, or conference proceedings were excluded. The systematic review was conducted according to the Joanna Briggs Institute Critical Appraisal Tools for the systematic review of texts and opinions (27).

### Search Strategy

Five databases were searched: Alt HealthWatch, Health Source—consumer edition magazines, Newspaper Source, Business Source Complete, and Academic Search. The searches included two concepts: raw or unpasteurized milk. The search was restricted to English Language reports and included all the states in the US and the Virgin Islands.

### Screening

Citations were uploaded to Rayyan, an application designed for sorting citations. The titles were screened, and those that seemed relevant were added to RefWorks and the full-texts were reviewed.

### Data Extraction

Equivalent information was extracted from all included reports. This information was comprised of the publication type; publishing regions [Federal Information Processing System (FIPS) 2015 codes were used to organize data by state and region/division]; the date, month, and year of publication; the category of opinions (supportive, against, or neutral); accessibility of information by the public (online, print, or both); and the frequency of publication (daily or monthly).

### Theoretical and Analytical Frameworks

In order to identify evidence gaps and future research needs, theoretical and analytical frameworks were designed and subsequently used to guide this review. For the current study, theoretical framework represents an explanation of the factors related to the likelihood of raw-milk acquired brucellosis while analytical framework is the visual representation of the complex factors associated with the increased prevalence of raw milk-acquired brucellosis in the United States.

### Analytic Framework of Direct and Indirect Measures

Google searches were used to identify direct and indirect measures of the elements identified in the analytical framework. Specifically, results were presented in a user-friendly format such as graphs and maps. Federal Information Processing System (FIPS) 2015 codes were used to organize data by state and region/division (28). FIPS grouped states into four regions with two or more divisions: Northeast (New England Division and Middle Atlantic Division); Midwest (East North Central Division and West North Central Division); South (South Atlantic Division, East South-Central Division, and West South-Central Division); and West (Mountain Division and Pacific Division). Maps were created using SPSS version 25.

Public opinions were coded as supportive, neutral, or against raw milk consumption. Within each state, an opinion message ratio was created by dividing the number of supportive/neutral messages within a state, by the total number of messages. Reported cases of brucellosis in the US (2012-2017) were obtained from the Centers for Disease Control and Prevention through the National Notifiable Diseases Surveillance System (NDSS).

### Statistical Analysis

Statistical analyses including univariate and bivariate analyses, as well as Chi-Square tests were conducted using the STATA statistical software (STATA, STATACorp LP, College Station, Texas, USA).

## Results

### Theoretical Framework

**Figure 1:**
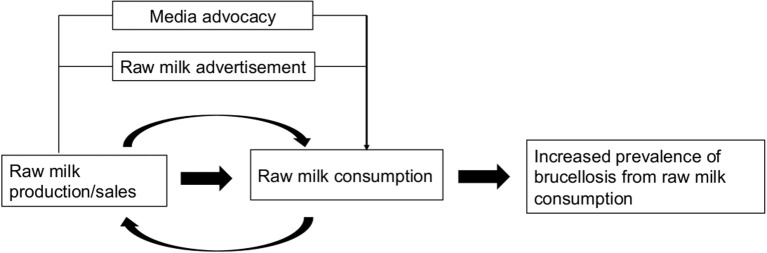
Theoretical framework of the factors related to the likelihood of raw-milk acquired brucellosis. An increase in raw milk sales and promotion through media advocacy could lead to increased purchase and consumption, ultimately leading to an increase of prevalence of raw milk-acquired brucellosis.

The theoretical framework that was used to guide this review is illustrated in Figure 1. We proposed that varying factors contribute to the prevalence of raw milk-acquired brucellosis. For example, raw milk sales promotion through advertisement and media advocacy could lead to the increased awareness of its availability for human consumption, as well as a surge in purchases, thereby leading to the increased prevalence of raw milk-acquired diseases such as brucellosis.

### Study Characteristics

**Figure 2:**
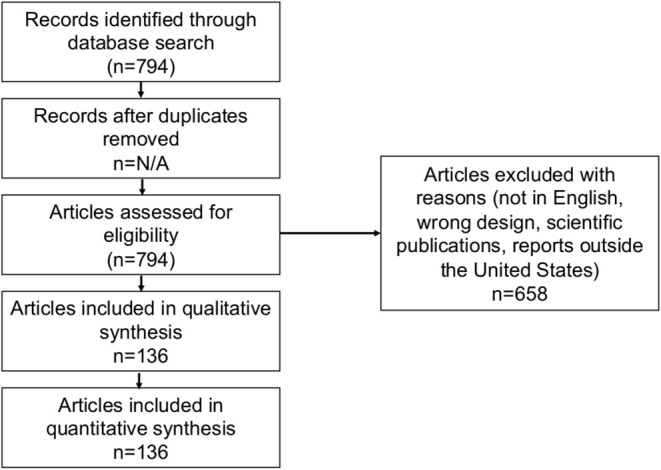
PRISMA flow chart of the systematic review of public opinions about raw milk consumption in the United States.

In this study, a total of 745 information sources were identified and analyzed for a qualitative systematic review. Figure 2 details the process of screening and selection of opinion messages, which was performed according to the Preferred Reporting Items for Systematic Review and Meta-Analyses guidelines (PRISMA) (29).

**Figure 3:**
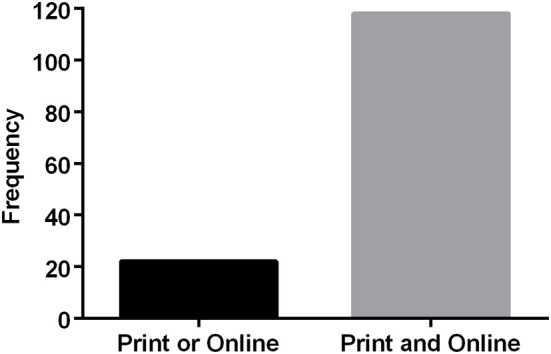
Accessibility of the public to the varying information sources that support or are against the consumption of raw milk.

One hundred and thirty-six opinion messages met the inclusion criteria, and they cut across 33 states including the District of Columbia. The publishing regions of the journals were grouped according to the US Census Bureau regional and divisional coding. The messages were coded (with respect to opinions about raw milk consumption) as “supportive (43/133),” “against (86/133),” or “neutral (4/133)” during the 6-year range 2012–2017 (each region or division was represented in the database with three cases of missing data for “state”). The majority of messages appeared in dual-format publications (print and online) (Figure 3), however we did not verify that within each of these dual-format publications any specific message did in fact appear in both formats. The vast majority (>80%) of publications had daily (sometimes twice a day) distributions with the remainder weekly, monthly, or quarterly. Most message authors contributed a message only once, and most publications also only contributed once.

### Data Analyses

The Federal Information Processing System (FIPS) coding, which comprises four US Census Bureau Regions was used in this current study. Each region or division was represented in the database with two cases of missing data for “state.” The (%) denotes the percentage representation of the 134 cases with state/FIPS identification, such that the sum of the division percentages within a region equal the region percentage (less rounding differences) (Table 1).

**Table 1:** Regional/divisional analyses.

**United States Regions**				**Milk production**	
	**State FIPS code**	**Number of cases**	**Percentage of cases (%)**	**Rank**	**Percentage (%)**
**Northeast**		**46**	**34.3**	**3**	**14**
*New England Division:*		*16*	*11.9*	*8*	*2*
Connecticut	9	1	0.7	34	0.2
Maine	23	12	9	33	0.3
Massachusetts	25	1	0.7	39	0.1
New Hampshire	33	0	-	37	0.1
Rhode Island	44	0	-	49	0.01
Vermont	50	2	1.5	17	1.3
*Middle Atlantic Division:*		*30*	*22.4*	*4*	*12*
New Jersey	34	1	0.7	44	0.1
New York	36	12	9	4	6.8
Pennsylvania	42	17	12.7	5	5.2
**Midwest**		**40**	**29.9**	**2**	**35.2**
*East North Central Division:*		*29*	*21.6*	*1*	*24.3*
Indiana	18	1	0.7	14	1.9
Illinois	17	7	5.2	22	0.9
Michigan	26	2	1.5	7	4.9
Ohio	39	1	0.7	11	2.6
Wisconsin	55	18	13.4	2	13.9
*West North Central Division:*		*11*	*8.2*	*5*	*11*
Iowa	19	1	0.7	12	2.3
Kansas	20	0	-	16	1.5
Minnesota	27	6	4.5	8	4.5
Missouri	29	2	1.5	25	0.7
Nebraska	31	0	-	26	0.6
North Dakota	38	1	0.7	35	0.2
South Dakota	46	1	0.7	20	1.1
**South**		**25**	**18.7**	**4**	**10.6**
*South Atlantic Division:*		*17*	*12.7*	*7*	*4.1*
Delaware	10	0	-	46	0.1
D.C.	11	1	0.7	-	-
Florida	12	0	-	18	1.2
Georgia	13	1	0.7	23	0.9
Maryland	24	2	1.5	29	0.5
North Carolina	37	0	-	28	0.5
South Carolina	45	0	-	38	0.1
Virginia	51	0	-	24	0.9
West Virginia	54	13	9.7	42	0.1
*East South-Central Division:*		*1*	*0.7*	*9*	*1*
Alabama	1	0	-	45	0.1
Kentucky	21	0	-	27	0.5
Mississippi	28	1	0.7	41	0.1
Tennessee	47	0	-	30	0.4
*West South-Central Division:*		*7*	*5.2*	*6*	*5.4*
Arkansas	5	0	-	47	0.04
Louisiana	22	2	1.5	40	0.1
Oklahoma	40	1	0.7	31	0.4
Texas	46	4	3	6	4.9
**West**		**23**	**17.2**	**1**	**40.2**
*Mountain Division:*		*10*	*7.5*	*3*	*16.2*
Arizona	4	0	-	13	2.3
Colorado	8	1	0.7	15	1.8
Idaho	16	0	-	3	6.8
New Mexico	35	1	0.7	9	3.8
Montana	30	5	3.7	36	0.1
Utah	49	1	0.7	21	1.1
Nevada	32	1	0.7	32	0.3
Wyoming	56	1	0.7	43	0.1
*Pacific Division:*		*13*	*9.7*	*2*	*24*
Alaska	2	0	-	50	0
California	6	5	3.7	1	19.6
Hawaii	15	0	-	48	0.02
Oregon	41	4	3	19	1.2
Washington	53	4	3	10	3.2

*The table denotes the number or percentage representation of the opinion messages included in the study [States (FIPS code) by Division (SPSS Code) by Region (SPSS Code)—not all states were represented in database]. Milk production was also ranked by states or regional division*.

*FIPS, Federal Information Processing Standard. The bold values represent the number of opinion messages per region; percentage of that per region; the rank of each region in milk production; and percentage of milk production per region*.

### Bivariate Analyses of Groupings

To determine the distribution of opinion messages by US regions, bivariate analysis of groupings was used. Opinion messages that indicated a support for or indifference about raw milk consumption were categorized as “Supportive” or “Neutral”, respectively, and those that were against raw milk consumption were categorized as “Against.” The region with the highest percentage of “against” messages (of messages within the region) was the West (91.3%), followed by the Midwest (69.2%), South (56.0%), and the Northeast (52.2%); chi-square = 11.461, 3 df, *p* = 0.009. The West region had the highest milk production and, among West messages, the highest percentage of “against” messages, but had the lowest percentage of total messages observed (Table 2).

**Table 2:** CenRegion crosstabulation.

		**CenRegion**				**Total (%)**
**Percentage (%) within CenRegion**						
		**Northeast (%)**	**Midwest (%)**	**South (%)**	**West (%)**	
Opinion	Neutral or Supportive	47.8	30.8	44.0	8.7	35.3
	Against	52.2	69.2	56.0	91.3	64.7
Total	100.0	100.0	100.0	100.0	100.0	
		**Value**	***df***	**Asymptotic significance (2-sided)**		
**Chi-Square Tests**						
Pearson chi-square		11.461^a^	3	0.009		
Likelihood ratio		13.057	3	0.005		
Linear-by-linear association		6.880	1	0.009		
N of valid cases		133				

a *0 cells (0.0%) have expected count <5. The minimum expected count is 8.13*.

### Univariate Analyses of Groupings

To determine the proportion of opinion messages that support or are against raw milk consumption, univariate analysis of groupings was used. Interestingly, the proportion of messages against raw milk consumption was higher than the messages that support it.

**Figure 4:**
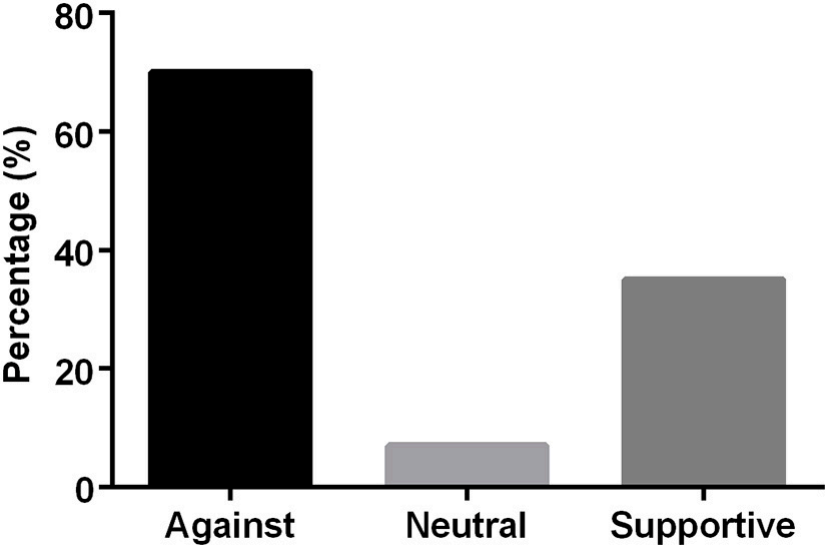
Sources of information categorized based on varying reactions to the consumption of raw milk in the United States. The graph represents the percentage of messages that are against, neutral or in support of the consumption of raw milk.

**Figure 5:**
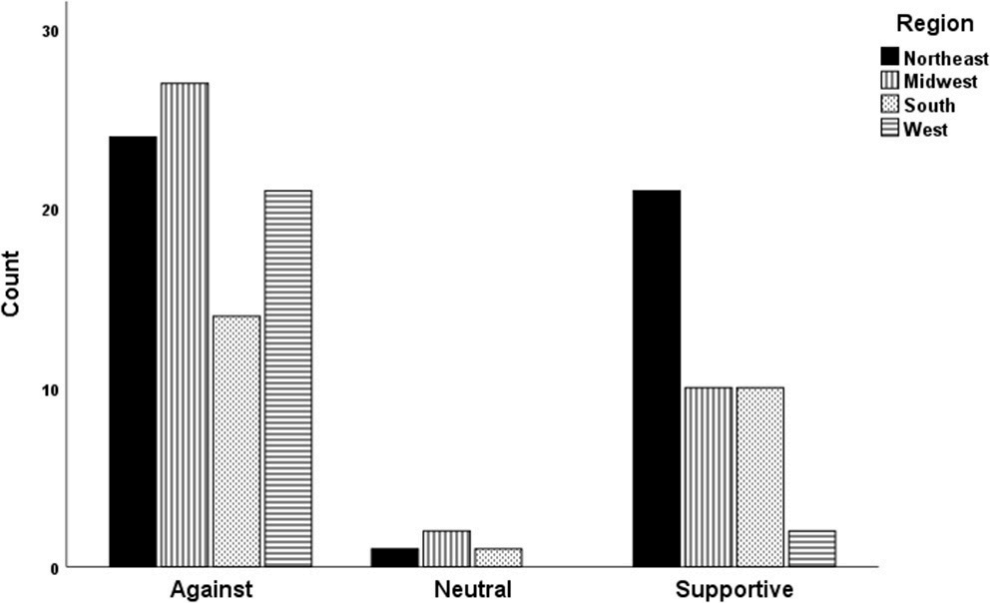
Public opinions categorized based on varying reactions to raw milk consumption in the United States. Neutral or Supportive: The graph represents the number of publications that were indifferent about or advocated the consumption of raw milk. Media from the Northeast had the most promotion of raw milk consumption.

However, the proportion of supportive messages appeared to be higher than those of neutral (Figure 4), indicating the trend and preference for raw milk consumption. In an attempt to characterize the regional distribution of the opinion messages, the messages were grouped based on the FIPS divisional coding as previously stated. The number of opinion messages that advocated raw milk consumption were highest in the Northeast (Figure 5).

### Confirmed Brucellosis Cases in the US

**Figure 6:**
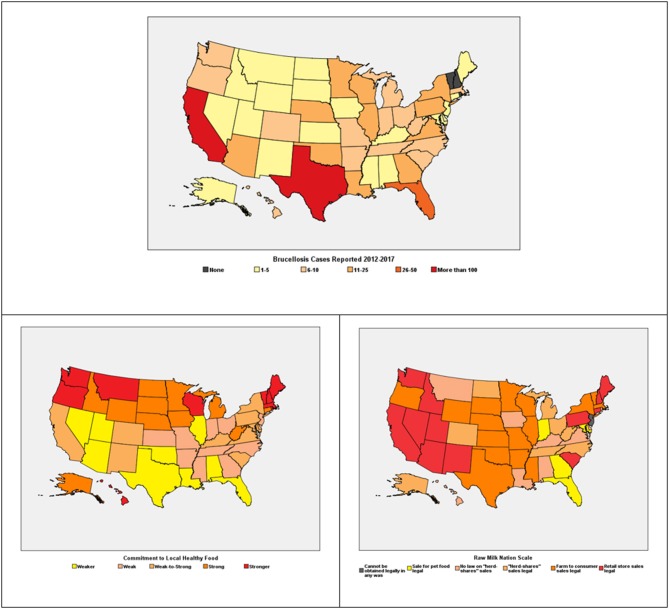
Number of confirmed cases of brucellosis in the US reported to the CDC. Outbreak of raw milk related diseases including brucellosis have been reported mostly in states that permit the sale of raw milk. Lovacore Index—A measure of the commitment of individuals to local healthy food. Legal status of the sale of raw milk in the United States.

Consumption of raw milk can lead to the acquisition of diseases that significantly impact the health of consumers, including brucellosis. Unfortunately, data demonstrating the proportion of raw milk-acquired brucellosis in the US is unavailable. Therefore, it was not possible to use this in further data analyses in the current study. However, for graphical representation, we used the confirmed cases of human brucellosis provided by the CDC, which represented the total number of cases irrespective of the source of acquisition. We found that brucellosis was also mostly reported in the states that had a high proportion of opinion messages supporting raw milk consumption (Figure 6). However, other factors that possibly contribute to the prevalence of brucellosis in some US states including immigration and close proximity to brucellosis-endemic countries like Mexico were not examined in this study.

### Analytical Framework

**Figure 7:**
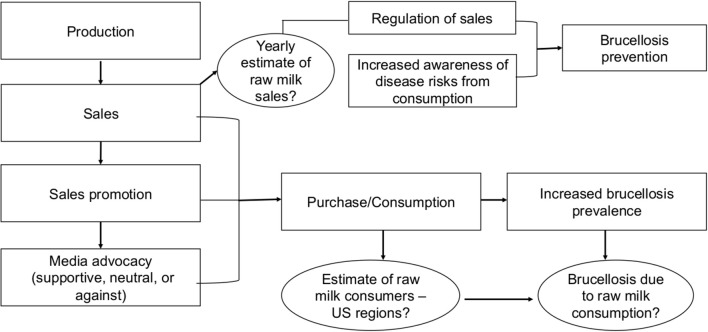
An analytical framework is proposed for understanding the complex factors associated with the increased prevalence of raw milk-acquired brucellosis in the United States. The circles represent priority areas and gaps in evidence that require additional research to facilitate the establishment of the safety and quality control measures for the raw milk sales process, and also design an effective strategy for the prevention of raw milk-acquired brucellosis.

In summary, the current study has identified several evidence gaps and factors that can possibly contribute to the increased prevalence of raw milk-acquired infectious diseases such as brucellosis (Figure 7). One of the primary goals of this study was to correlate raw milk supportive messages with increased sales and purchases of raw milk as well as the increased prevalence of raw milk-acquired brucellosis in US regions. Unfortunately, the conclusions from this study have been limited by the inaccessibility of pertinent data such as an estimate of regional or national raw milk sales, demographics of consumers, and particularly, cases of human brucellosis resulting from raw milk consumption. Availability of these data will facilitate efforts to design an effective strategy for the prevention of raw milk-acquired brucellosis through the regulation of sales, increased awareness of disease risks associated with consumption, and the establishment of safety and quality control measures.

## Discussion

The scientific evidence of the health risks associated with the consumption of raw milk and products has been known for a long time (30, 31). However, less clear is the impact of acquiring infectious diseases including brucellosis from raw milk consumption in the US due to incomplete reporting of cases and the complex factors associated with the sale and consumption of raw milk, including inconsistent policies that range from total prohibition to legal sales in retail stores. One of the aims of this current study was to determine if increase in sales and consumption of raw milk in the US is directly associated with increased media advocacy and public opinions about the benefits of consuming raw milk. Interestingly, we found that the majority of public opinion published by newspapers and magazines in the Northeastern, Midwestern, Southern, and Western regions of the US were against the sale and consumption of raw milk due to the associated health risks. Hence, the rise in the trend of raw milk consumption may be a result of other factors such as the dissemination of misleading information that are neither evidence nor science-based on other social media networks like Facebook, Twitter, and other social interactive platforms. Previous studies have shown that discussions on these types of social platforms have severe implications in influencing consumer behaviors (32).

Another important finding in this current study was that majority of the media advocacy and public opinion in favor of raw milk consumption were published in the Northeast compared to other US regions. Why the Northeast had more favorable public opinion is not known, but it may be due to a stronger commitment of individuals in this US region to healthy local foods as indicated by Lovacore Index which ranks states based on the support of natural products or food (Figure 6). Additionally, the favorable raw milk regulations in the Northeast also facilitates the ease of access to raw milk via various means including availability in retail stores and farms where raw milk is produced (Figure 6). Moreover, media advocacy and public opinion were also accessible via both printed and online, making it possible to reach a larger audience. Therefore, efforts should be intensified for the adoption of media advocacy as well as social networks to increase awareness and educate the public about the disease risks associated with raw milk consumption. It is important to bear in mind that the more favorable public opinion in the Northeast does not directly correlate with increased incidence of brucellosis when compared to other regions like the southern states. A possible explanation for this might be the interplay of factors that contribute to disease incidence and prevalence in different regions including immigration and interaction with wild animals that serve as reservoir hosts.

What prompted the investigation of this current study were the recent increase in the number of confirmed human brucellosis cases resulting from the consumption of raw milk and products. Interestingly, human brucellosis is an almost nonexistent disease in the US, but endemic in countries where the consumption of raw milk is greatest and unregulated. Symptoms in infected individuals are non-specific and can include fever, sweats, arthralgia, myalgia, and in complicated cases, miscarriage or spontaneous abortion (1, 33–35). In this study, an attempt to directly correlate confirmed human brucellosis cases with the consumption of raw milk and products was impossible, which demonstrates a gap in evidence of unavailable data reporting human brucellosis acquired from raw milk consumption. Therefore, we cannot prove that raw milk consumption contributed to increased prevalence of brucellosis in the US. It is notable that apart from raw milk consumption, there are several other factors that increase the risk of acquisition of human brucellosis including occupations that allow direct contact with animals (e.g., veterinarians, butchers, ranchers, animal care givers, etc.), laboratory personnel or research scientists that have direct exposure to animal samples, immigration, as well as feral swine hunting that results in species-specific infections. An important issue for future research is to unravel how raw milk consumption specifically contributes to the prevalence of brucellosis in the US. However, in endemic regions, raw milk consumption accounts for the most common cause of human brucellosis (36, 37). For this study, the number of brucellosis cases publicly provided by the CDC did not delineate the cases based on the source of infection. Therefore, the ease of access to cases of raw milk-acquired brucellosis is paramount to further generate effective brucellosis control and prevention strategies so as to avoid a backward trend of disease outbreaks due to raw milk consumption that occurred in the 1900s. Moreover, in order to reduce the incidence of raw milk-acquired brucellosis, several measures must be implemented including but not limited to promoting pasteurization, restricting the sale and access to raw milk, establishing rigorous quality control of the raw milk sale process, and the development of diagnostic tests for validating the safety of raw milk. This is crucial due to the fact that no diagnostic tests are available or approved for raw milk and milk products in the US or globally (US—Food and Drug Administration). Because of the current difficulty in restricting the sale of and access to raw milk, a better approach to limiting the associated disease risks may be an effective regulation of the quality of raw milk provided for human consumption, which will involve integrated efforts from veterinary services, regulations from the Federal Department of Agriculture (FDA), as well as effective training of physicians, farmers, ranchers, and consumers.

To facilitate informed policy decisions about restricting the sale and consumption of raw milk and products in the US, and ultimately reduce the risk of raw milk acquired diseases including brucellosis, an analytical framework was proposed (Figure 7). One of the research priority areas identified was the lack of regional or nation-wide data reporting the sales and purchases of raw milk in the US. This lack of raw milk sales data precluded the probable analyses and conclusions that may demonstrate the direct association of the magnitude of sales and purchases of raw milk with an increased brucellosis prevalence. We argue that data reporting the US regional or state-wide raw milk sales will help to establish the influence of the sales process on disease prevalence in the country. In addition, information about the purchases and demographics of raw milk consumers will help to further understand consumer attitude and behavior toward the consumption of raw milk, and also help to increase awareness about the potential risks of raw-milk acquired diseases. In other words, to formulate policies on the reduction of raw milk sales and distribution, the impact of raw milk production, sales promotion, consumer attitudes, and behavioral patterns must all be critically evaluated (12).

Additionally, we propose that raw milk sales promotion may influence consumer behavior and motivation by contributing to increased awareness of the availability of raw milk for purchases. Previous studies have shown that the purchase and consumption of raw milk are not restricted to a particular age group, income, education level of consumers, or distance to the place of purchase. In fact, in these studies, there was no association between these factors and raw milk consumption (21). Therefore, sales promotion, possibly through social media networks, likely presents a huge influence on consumer attitude and motivation for raw milk consumption.

The findings in this current study have demonstrated that media advocacy and public opinion possibly contribute to increased trend of raw milk consumption in different US regions, and that several factors might be involved in the prevalence of raw milk-acquired diseases like brucellosis. Additionally, the evidence gaps identified in this study have provided a strong basis for future investigations and the development of effective strategies to alleviate the risks associated with raw milk-acquired infectious diseases including brucellosis. This will help to prevent outbreaks of human brucellosis in the US, which can have both direct and indirect implications including increased healthcare costs and potential threats to food safety and security due to the loss of livestock production. Evidence-informed health policies are most effective when guided by science, consumer preferences, and political reality. Hence, we strongly recommend an interdisciplinary approach and effort toward the building of the raw milk consumption evidence base.

## Data Availability Statement

All datasets generated for this study are included in the article/Supplementary Material.

## Author Contributions

SA, AF, GR, and AA-G: conceptualization. SA, MF, and GR: data curation. SA, GR, and LP: formal analysis. SA, GR, and AA-G: investigation. SA, GR, MF, and LP: methodology. SA, AF, MF, LP, and AA-G: resources. AA-G: supervision. SA, GR, and AA-G: writing—original draft. SA, AF, MF, LP, and AA-G: writing—review & editing.

### Conflict of Interest

AF is a managing partner of NanoRelease Technologies (NRT), LLC Inc., has a 95% equity interest in NRT, a company involved in vaccine delivery platforms. The terms of this arrangement have been reviewed and approved by TXAgriLife Research and Texas A&M University in accordance with their conflict of interest policies. The remaining authors declare that the research was conducted in the absence of any commercial or financial relationships that could be construed as a potential conflict of interest.

## References

[1] Pappas G Papadimitriou P Akritidis N Christou L Tsianos EV. The new global map of human brucellosis. Lancet Infect Dis. (2006) 6:91–9. 10.1016/S1473-3099(06)70382-6 16439329

[2] Kiel FW Khan MY. Brucellosis in Saudi Arabia. Social Sci Med. (1989) 29:999–1001. 2814586 10.1016/0277-9536(89)90056-7

[3] Seleem MN Boyle SM Sriranganathan N. Brucellosis: a re-emerging zoonosis. Vet Microbiol. (2010) 140:392–8. 10.1016/j.vetmic.2009.06.021 19604656

[4] Christie A Brucellosis, undulant fever, Malta or Mediterranean fever. In: Christ A editor. Infectious Disease: Epidemiology and Clinical Practice. 3rd ed. London: Churchill Livingstone (1980). p. 824–47.

[5] Bruce D discoverer of brucellosis. Singapore Med J. (2011) 52:138. 21451919

[6] Langer AJ Ayers T Grass J Lynch M Angulo FJ Mahon BE. Nonpasteurized dairy products, disease outbreaks, and state laws—United States, 1993–2006. Emerging Infect Dis. (2012) 18:385. 10.3201/eid1803.111370 22377202 PMC3309640

[7] Colmenero JD Reguera J Martos F Sanchez-De-Mora D Delgado M Causse M. Complications associated with *Brucella melitensis* infection: a study of 530 cases. Medicine. (1996) 75:195–211. 10.1097/00005792-199607000-00003 8699960

[8] Pourbagher A Pourbagher MA Savas L Turunc T Demiroglu YZ Erol I. Epidemiologic, clinical, and imaging findings in brucellosis patients with osteoarticular involvement. Am J Roentgenol. (2006) 187:873–80. 10.2214/AJR.05.1088 16985128

[9] Guler S Kokoglu OF Ucmak H Gul M Ozden S Ozkan F. Human brucellosis in Turkey: different clinical presentations. J Infect Dev Ctries. (2014) 8:581–8. 10.3855/jidc.3510 24820461

[10] Al Dahouk S Nöckler K Hensel A Tomaso H Scholz HC Hagen RM. Human brucellosis in a nonendemic country: a report from Germany, 2002 and 2003. European J Clin Microbiol Infect Dis. (2005) 24:450–6. 10.1007/s10096-005-1349-z 15959815

[11] Buzby JC Hannah GL Kendall ME Jones TF Robinson T Blayney DP Characteristics of consumers of unpasteurized milk in the United States. J Consumer Affairs. (2013) 47:153–66. 10.1111/joca.12001

[12] Kearney J 2010. Food consumption trends and drivers. Philos Trans R Soc B Biol Sci. (2010) 365:2793–807. 10.1098/rstb.2010.0149 PMC2935122 20713385

[13] Oliver SP Jayarao BM Almeida RA. Foodborne pathogens in milk and the dairy farm environment: food safety and public health implications. Foodbourne Pathog Dis. (2005) 2:115–29. 10.1089/fpd.2005.2.115 15992306

[14] Angulo FJ LeJeune JT Rajala-Schultz PJ Unpasteurized milk: a continued public health threat. Clin Infect Dis. (2009) 48:93–100. 10.1086/595007 19053805

[15] Headrick ML Korangy S Bean NH Angulo FJ Altekruse SF Potter ME. The epidemiology of raw milk-associated foodborne disease outbreaks reported in the United States, 1973 through 1992. Am J Public Health. (1998) 88:1219–21. 10.2105/AJPH.88.8.1219 9702153 PMC1508307

[16] Leamy RJ Heiss SN Roche E The impact of consumer motivations and sources of information on unpasteurized milk consumption in Vermont, 2013. Food Protect Trends. (2013) 34:216–25.

[17] Cossaboom CM Kharod GA Salzer JS Tiller RV Campbell LP Wu K. Notes from the field: *Brucella abortus* vaccine strain RB51 infection and exposures associated with raw milk consumption—Wise County, Texas, 2017. Morb Mortal Wkly Rep. (2018) 67:286. 10.15585/mmwr.mm6709a4 29518066 PMC5844281

[18] Sfeir MM. Raw milk intake: beware of emerging brucellosis. J Med Microbiol. (2018) 67:681–2. 10.1099/jmm.0.000722 29537364

[19] Negrón ME Kharod GA Bower WA Walke H. Notes from the Field: Human *Brucella abortus* RB51 infections caused by consumption of unpasteurized domestic dairy products—United States, 2017–2019. Morb Mortal Wkly Rep. (2019) 68:185. 10.15585/mmwr.mm6807a6 30789879 PMC6385706

[20] Steele JH. History, trends, and extent of pasteurization. J Am Vet Med Assoc. (2000) 217:175–8. 10.2460/javma.2000.217.175 10909453

[21] Katafiasz AR Bartlett P Motivation for unpasteurized milk consumption in Michigan, 2011. Food Prot Trends. (2012) 32:124–8.

[22] Mungai EA Behravesh CB Gould LH. Increased outbreaks associated with nonpasteurized milk, United States, 2007–2012. Emerg Infect Dis. (2015) 21:119. 10.3201/eid2101.140447 25531403 PMC4285278

[23] Rahn WM Gollust SE Tang X Framing food policy: the case of raw milk. Pol Stud J. (2017) 45:359–83. 10.1111/psj.12161

[24] Alvarez VB Parada-Rabell F Health benefits, risk and regulations of raw and pasteurizes milk. Factsheet Extension. (2005) 3:5.

[25] Potter ME Kaufmann AF Blake PA Feldman RA. Unpasteurized milk: the hazards of a health fetish. JAMA. (1984) 252:2048–52. 6481912 10.1001/jama.252.15.2048

[26] Rogers EM (1962). Diffusion of Innovations. New York, NY: Free Press.

[27] McArthur A Klugarova J Yan H Florescu S. Innovations in the systematic review of text and opinion. Int J Evid Based Health. (2015) 13:188–95. 10.1097/XEB.0000000000000060 26207851

[28] FIPS Codes United States Census and Bureau. Available online atL https://www.census.gov/geographies/reference-files/2015/demo/popest/2015-fips.html

[29] Moher D Liberati A Tetzlaff J Altman DG. Preferred reporting items for systematic reviews and meta-analyses: the PRISMA statement. Ann Internal Med. (2009) 151:264–9. 10.7326/0003-4819-151-4-200908180-00135 19622511

[30] Abbas BA Aldeewan AB Occurrence and epidemiology of *Brucella* spp. in raw milk samples at Basrah province, Iraq. Bulgarian J Vet Med. (2009) 12:2.

[31] Rijpens NP Jannes G Van Asbroeck MA Rossau R Herman LM. Direct detection of Brucella spp. in raw milk by PCR and reverse hybridization with 16S-23S rRNA spacer probes. Appl Environ Microbiol. (1996) 62:1683–8. 8633866 10.1128/aem.62.5.1683-1688.1996 PMC167942

[32] Schmidt AL Zollo F Scala A Betsch C Quattrociocchi W. Polarization of the vaccination debate on Facebook. Vaccine. (2018) 36:606–12. 10.1016/j.vaccine.2018.05.040 29773322

[33] Young EJ. An overview of human brucellosis. Clin Infect Dis. (1995) 21:283–9. 8562733 10.1093/clinids/21.2.283

[34] Khan MY Mah MW Memish Z.A. 2001. Brucellosis in pregnant women. Clin Infect Dis. (2001) 32:1172–7. 10.1086/319758 11283806

[35] Arenas-Gamboa AM Rossetti CA Chaki SP Garcia-Gonzalez DG Adams LG Ficht TA. Human brucellosis and adverse pregnancy outcomes. Curr Trop Med Rep. (2016) 3:164–72. 10.1007/s40475-016-0092-0 29226068 PMC5720386

[36] Lulu AR Araj GF Khateeb MI Mustafa MY Yusuf AR Fenech FF. Human brucellosis in Kuwait: a prospective study of 400 cases. QJM. (1988) 66:39–54. 3051080

[37] Ramos JM Bernal E Esguevillas T Lopez-Garcia P Gaztambide MS Gutierrez F. Non-imported brucellosis outbreak from unpasteurized raw milk in Moroccan immigrants in Spain. Epidemiol Infect. (2008) 136:1552–5. 10.1017/S0950268807000210 18205974 PMC2870757

